# Medical students respond to the call to climate action

**DOI:** 10.1016/j.joclim.2025.100439

**Published:** 2025-04-15

**Authors:** Aroub Khan Yousuf, Torey Katzmeyer, Harleen K Marwah

**Affiliations:** Harvard Medical School, 25 Shattuck St, Boston, MA, 02115, USA; West Virginia University School of Medicine, 64 Medical Center Dr, Morgantown, WV, 26506, USA; c Children's Hospital of Philadelphia, United States

Climate change is a public health emergency, and it is critical that physicians-in-training are prepared to provide care for climate-related conditions and diseases [[1], [2], [3], [4]].

In *Perspectives on climate change in medical school curricula—A survey of* U*.S. medical students*, 83.9 % of 600 medical students across 12 medical schools reported they believe that climate health should be a focus of their medical school curricula [5]. According to *A qualitative study of what motivates, facilitates, and hinders climate-engaged healthcare trainees to advance healthcare sustainability*, healthcare trainees engaged in healthcare sustainability projects are specifically motivated to address healthcare sustainability through “positive role models, the health impacts of climate change, observation of unsustainable healthcare practices, and a sense of social responsibility” [6]. Hence, many medical students are already prioritizing climate health and action as part of their medical school experience. We seek to underscore the importance of training medical students in the fight against climate change, the first step of which is inclusion of climate health across medical school curricula nationally. We believe that it is essential for future physicians to be able to identify and counsel patients at higher risk, while utilizing their platforms as trusted community members to raise awareness about the health consequences of the climate crisis.

Medical schools focus on identification of symptoms and prescribing solutions, for example, inhalers for asthma, antimicrobials for vector-borne infections, and selective serotonin reuptake inhibitors for mental illnesses. In the wake of climate change, we recognize we may only be putting bandages on a larger problem hurting our patients; we need solutions that target climate-related disease before the development of these devastating consequences on human health and our environment.

Medical Students for a Sustainable Future (MS4SF) addresses these issues head on. MS4SF is a national network of medical students who recognize the health implications of climate change and acts through research, medical education, advocacy, and sustainability efforts. Nearly 1,000 students from over 100 schools have joined the organization in the past five years. The MS4SF community knows that human health cannot be optimized without educating healthcare trainees and active efforts to prevent the health impacts of climate change.

Here are some steps that medical students and other health professionals can take:

1) Work with your medical school administration and professors to start or add to a climate health curriculum. Reference *MS4SF's Guide to Climate Health and Curriculum Reform* to learn more [7].
2) Join sustainability initiatives at your school and/ or hospital.
3) Work with local policymakers to both support and draft legislation in favor of healthy, sustainable communities. As a healthcare professional, you are a trusted member of your community, and your opinion matters.
4) Join an initiative such as MS4SF and consider attending MS4SF's annual Climate Health Equity Day, an in-person and online event intended to bring together leaders in climate health.
5) Work alongside grassroots organizations which are fighting against climate change and pollution.
6) Educate your patients and colleagues on the relationship between environmental degradation and human health. Some resources are provided by Harvard T.H. Chan School of Public Health [8].

The consequences of the climate crisis on human health cannot be overstated. We see first-hand that the health of our own communities is negatively affected by climate change. Through patient experiences, we are constantly reminded that the well-being of humanity is being threatened. *Perspectives on climate change in medical school curricula—A survey of* U*.S. medical students* highlighted the desire of medical students to learn more about climate health. Thus, we urge medical schools to adopt a climate health curriculum to educate future doctors on the impact of environmental degradation on human health and train medical students in climate health advocacy.

## All co-authors

Aroub Yousuf, Harvard Medical School

Torey Katzmeyer, West Virginia University School of Medicine

Harleen K Marwah, Children's Hospital of Philadelphia

## CRediT authorship contribution statement

**Aroub Khan Yousuf:** Writing – review & editing, Writing – original draft, Conceptualization. **Torey Katzmeyer:** Writing – review & editing, Writing – original draft, Conceptualization. **Harleen K Marwah:** Writing – review & editing, Conceptualization.

## Declaration of competing interest

The authors declare the following financial interests/personal relationships which may be considered as potential competing interests:

All authors are board members of Medical Students for a Sustainable Future (MS4SF).
